# Methylation and Expression of the Exercise-Related TLR1 Gene Is Associated With Low Grade Glioma Prognosis and Outcome

**DOI:** 10.3389/fmolb.2021.747933

**Published:** 2021-11-16

**Authors:** Lichun Lu, Yifang Hu, Chen Wang, Feng Jiang, Chuyan Wu

**Affiliations:** ^1^ Department of Neurology, The Affiliated Suzhou Science & Technology Town Hospital of Nanjing Medical University, Suzhou, China; ^2^ Department of Geriatrics, The First Affiliated Hospital of Nanjing Medical University, Nanjing, China; ^3^ Department of Neonatology, Obstetrics and Gynecology Hospital of Fudan University, Shanghai, China; ^4^ Department of Rehabilitation Medicine, The First Affiliated Hospital of Nanjing Medical University, Nanjing, China

**Keywords:** exercise-related genes, methylation, prognosis, immune cell infiltration, low grade glioma

## Abstract

**Background:** Exercise improves function, reduces disability, maintains independence, and improves quality of life for low-grade glioma (LGG) patients. Exercise can also improve the effectiveness of cancer treatment. The goal of this research was to find potential exercise related genes that may be used to predict exercise levels and may be used as a biomarker for cancer outcomes.

**Methods:** The GSE111551 database was thoroughly examined in this research, and the resulting conclusion of exercise-related genes was reached. The protein interaction network (PPI) was used to examine the differentially expressed genes (DEGs). Then the exercise-related gene TLR1 was chosen. The expression, methylation degree, prognosis, and immune relevance of TLR1 were investigated using bioinformatics. In addition, we verified the role of TLR1 in Glioma cell lines.

**Results:** LGG patients with reduced TLR1 expression and hypermethylation had a better overall survival (OS) and progression free survival (PFS), using the TCGA database. Low TLR1 expression and hypermethylation of TLR1 were found to be independent biomarkers for OS using Cox regression. Furthermore, the CGGA database was used to confirm the prognostic function of TLR1 in this cancer. Finally, most methylation sites of TLR1 were strongly correlated with immune infiltration and immune checkpoint. Then, reducing TLR1 expression substantially slowed the cell cycle and decreased LGG cell proliferation, emigration, and infiltration *in vitro*.

**Conclusions:** Exercise-related gene TLR1 has the potential to be a useful prognostic biomarker, and it is thought to be involved in immune cell infiltration and immunotherapy in LGG.

## Introduction

Tumors derived from the neuroepithelium are called gliomas, accounting for 29% of the central nervous system cancers, which is the most common primary intracranial tumor (Du et al., 2021). The most prevalent identified malignant cancer in the brain is low-grade glioma, which has a large degree of inherent heterogeneity in terms of tumor biological conduct (Ostrom et al., 2013). Despite extensive treatment for LGG, which includes neurosurgical resection, chemotherapy, and radiotherapy, therapeutic resistance and tumor recurrence appear to be unavoidable (Cancer Genome Atlas Research Network et al., 2015). Any LGG patients are slow to improve, whereas others develop high-grade glioblastoma, which has a poor prognosis (Tan et al., 2020).

**GRAPHICAL ABSTRACT:**
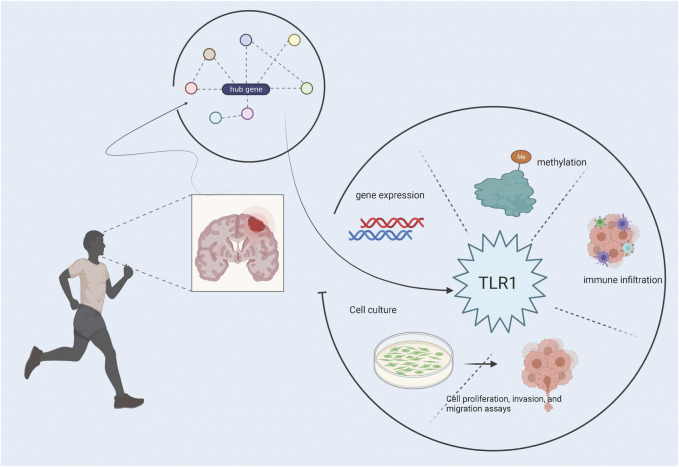
Exercise-related TLR1 gene plays a certain role in the prognosis judgment and immunotherapy of LGG.

Exercise has a strong theoretical basis for being an effective intervention for managing symptoms of brain cancer and treating side effects (Schmidt et al., 2015; Nelson, 2016). Currently, animal experiments and clinical experiments have confirmed the encouraging relationship between appropriate physical exercise and improving the survival results of patients with malignant tumors of the nervous systemanimal (Carson et al., 2007; Wu et al., 2010; Cormie et al., 2015; Tantillo et al., 2020). As a consequence, the voluntary physical exercise program can be used in the therapeutic environment as an auxiliary and non-invasive therapy for glioma, enhancing patient’s quality of life. In other cancer patients and chronic patients, clinical studies also have confirmed the efficacy of appropriate exercise in combating physical impairment, cognitive impairment, and psychological effects such as depression and anxiety (Cormie et al., 2015). However, more research is needed to determine the best exercise treatment plan for these patient’s specific needs in order to improve protection and efficacy. When recommended and monitored properly, the possible rehabilitation impact of selected exercise intervention in neurooncology is impressive, particularly because exercise is a reasonably inexpensive and simply to access medication with little adverse side effects.

Toll-like receptors (TLRs) are a kind of non-specific immunity (innate immunity) protein molecule that also serves as a link between non-specific and specific immunity. The protein encoded by TLR1 gene is a member of the Toll-like receptor (TLR) family, which is widely expressed at higher levels than other TLR genes. TLR1 was identified as a TLR member that recognizes triacyl lipopetides; in TLR1 knockout mice, macrophages produced less inflammatory cytokine in response to triacyl lipopetides and lipoproteins from mycobacteria (Yu et al., 2021). It was verified in an animal experiment that TLR1 is expressed in mouse brain neurons, astrocytes, and microglia and may be implicated in the development of epilepsy (Wang et al., 2015). The link between TLR1 and exercise has been studied in a limited number of studies. TLR1 expression was considerably lower after post-acute exercise and after 2 h recovery compared to samples obtained at rest, according to relevant studies (Lancaster et al., 2005; Gleeson et al., 2006). However, studies on the link between long-term exercise and TLR1 expression are currently lacking.

**FIGURE 1:**
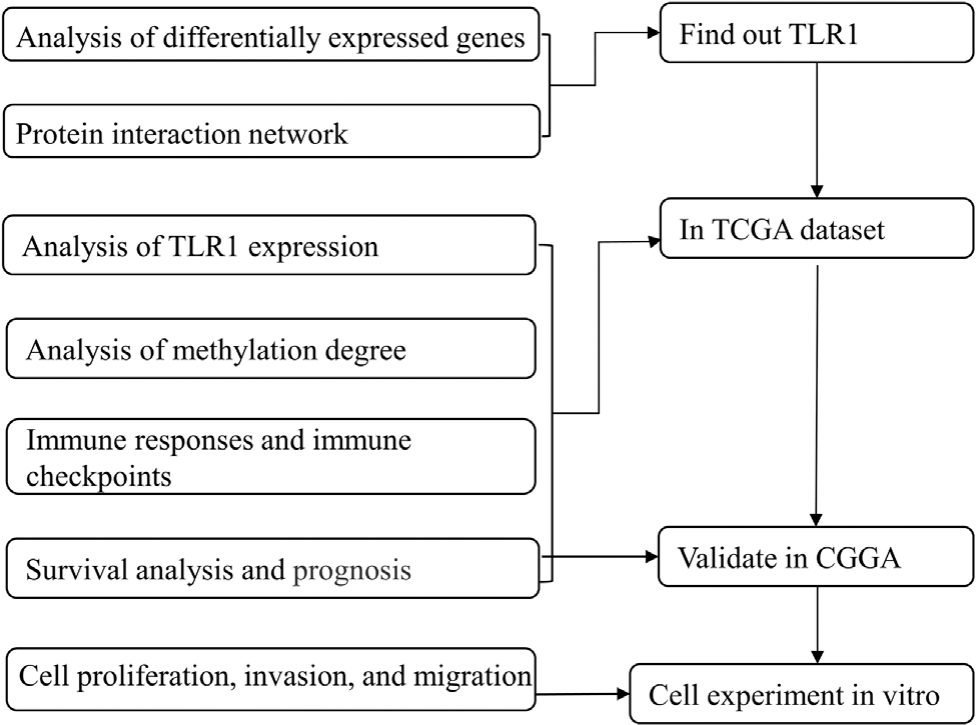
Flow progress diagram.

To find genes associated with exercise, we used the GSE111551 dataset from the Gene Expression Omnibus (GEO) database (Hao et al., 2021). We discovered genes that are differentially expressed after exercise. The gene expression level, methylation level, and clinicopathological features of TLR1 were investigated using the Cancer Genome Atlas (TCGA) database. Our findings indicate that TLR1, which is linked to exercise, can play a role in the production and incidence of LGG. It maybe plays a certain role in the prognosis judgment and immunotherapy therapy of gliomas (Graphic Abstract and Figure 1).

## Materials and Methods

### Processing of Data and Network of Protein-Protein Interactions Analysis

The GEO database was used to obtain GSE111551. The transcriptional histograms of 13 healthy male subject’s before and after exercise (running, 3 times/wk, 60min, 18w) were found in the database. LGG patient’s clinical information and transcriptional profiles were then downloaded from the TCGA (Gao et al., 2013) and CGGA database (Hu et al., 2018) (http://www.cgga.org.cn/). The protein-protein interaction (PPI) network was utilized to find key genes and gene modules associated with exercise. The Search Tool for the Retrieval of Interacting Genes (STRING) database was used to build the PPI network (http://www.stringdb.org/). Then, using the online resource Gene Expression Profiling Interactive Analysis (GEPIA) (http://gepia. cancer-pku. cn/index.html), we evaluated the differences in TLR1 mRNA expression in normal and LGG tissues (Tang et al., 2017).

### Methylation Site Analysis and Correlation Comparison With Expression

We downloaded the methylation profiles of patients with LGG from TCGA database via the Cbioportal website (https://www.cbioportal.org/). We analyzed the CpG sites of TLR1 gene. Then, using Pearson correlation analysis, the TLR1 CpG sites whose methylation was most significantly associated with TLR1 mRNA expression were identified.

### Survival Analysis of Gene Expression and Methylation Sites Using TCGA-Low-Grade Glioma Dataset

The Kaplan-Meier survival analysis was used to demonstrate the survival link between the high-risk and low-risk groups. In the experiment, the gene expression and prognosis of different groups were examined using heatmap and scatter dot plot. We evaluated the ROC curve’s predictive abilities in order to determine the prediction’s accuracy. Correlation analysis was used to establish relationships between the risk score and the patient’s clinicopathological variables. To validate the independent prediction model, we have utilized univariate and multivariate Cox regression analyses.

### Validation of Prognostic Significance in CGGA and Creation of a Projected Nomogram

We used univariate and multivariate Cox regression analysis to verify the prediction effect in CGGA database. In order to provide a valid clinical standard measure for LGG patients in terms of 1-, 3-, and 5-years survival, a nomogram based on risk values as well as other clinicopathological characteristics was developed. The calibration curves were then utilized for assessing the concordance between patients anticipated and observed.

### Relationship Between Gene Expression, Methylation Level and Immune Infiltration

CIBERSORT(Newman et al., 2015; Charoentong et al., 2017), ESTIMATE (Yoshihara et al., 2013), MCPcounter (Shi et al., 2020), TIMER algorithms (Li et al., 2017), QUANTISEQ (Finotello et al., 2019), EPIC (Racle et al., 2017), and XCELL (Aran et al., 2017) were evaluated to measure cellular function or immune effects between low and high risk groups according to TLR1 expression. A Heatmap was used to reveal variations in immune reaction under various algorithms. Furthermore, single-sample gene set enrichment analysis (ssGSEA) (Yi et al., 2020) was utilized to compare and identify the tumor-infiltrating immune cell subsets. Previous literature also yielded a possible immunological check-point.

### Biological Process Analysis

The TCGA dataset had two types of glioma patients: those with belevated TLR1 expression and those with low expression. With a false discovery rate of less than 0.05, the genes that varied in expression between the two groups were selected. Gene terms with a *p* value less than 0.01 and |logFC|≥1 were deemed important. The role of TLR1 in glioma was then investigated using GO analysis.

### Cell Culture and Transfection

Glioma cell lines (NHA, A172, U87, U251, T98, LN229) were acquired from the cell bank of the Chinese Academy of Sciences (Shanghai). RPMI 1640 (Gibco, Gaithersburg, MD, United States) was cultured with 10% fetal bovine serum (HyClone, Logan, United States) and 1% penicillin/streptomycin (Gibco) in an incubator at 37°C and 5% CO_2_. Transfections were performed applying OPTI-MEM (Invitrogen) and Lipofectamine 3000 by the manufacturer’s instructions. The siTLR1-1 (5′- AAC​ACA​ACT​AAC​TAC​AGA​TTA​CA -3′), siTLR1-2 (5′-ACU​GAU​AUC​AAG​AUA​CUG​GAT-3′) and siNC were bought from Tsingke (Nanjing, China) and introduced into cells at a concentration of 50 nM. The transfected cells were harvested at 24 h after transfection.

### Cell Proliferation, Invasion, and Migration Assays

The Cell Counting Kit-8 (CCK-8) and colony formation assays were applied to explore the ability of proliferation of cancer cells in different groups. In CCK-8 experiment, a total of 2,500 cancer cells were added into each well of 96-well plate. 10μL of CCK-8 solution (Dojindo Laboratiories, Kumamoto, Japan) was added into 96-well, then the absorbance of each well was analyzed at 450 nm after an incubation at 37°C for 2 h. For colony formation experiment, 1,000 cells of different groups were added into each well of a six-well plate. The culture medium was changed every 72 h. Crystal violet and 4% paraformaldehyde were applied to stain and fix the cells when the appearance of colonies could be recognized. The wound healing and transwell assays were applied to explore the ability of cellular migration and invasion.

### Western Blotting Analysis

After being washed using PBS, the cells were lysed with RIPA (radioimmunoprecipitation assay) solution containing a protease inhibitor. The acquired proteins were then quantified using a bicinchoninic acid protein assay (BCA) kit, after which 20 mg of total protein was separated on 8% or 10% sodium dodecyl sulfate polyacrylamide gel (SDS-PAGE) under electric field. The separated proteins were blotted onto a polyvinylidene difluoride (PVDF) membrane (Millipore, United States), which was then blocked with 5% bovine serum albumin (BSA) solution for 1 h and incubated with primary antibodies against GAPDH (1:1,000, Proteintech, United States), RBM15 (1:1,000, Proteintech, United States), METTL14 (1:1,000, Proteintech, United States), PDCD1 (1:2,000, Proteintech Group, United States), CTLA4 (1:1,000, novus biotechnologies, United States), HNRNRC (1:1,000, abcam, United States), and BTLA (1:1,000, abcam, United States) overnight at 4°C. On the second day, the membrane was incubated with secondary antibodies (1:2,500) at room temperature for 1 h after three times of washes with TBST (Tris-buffered saline-Tween). After being washed for another three times, bands of conjugate proteins were visualized via a ChemiDoc™ MP Imaging system (Bio-Rad Laboratories) with GADPH as the internal control.

### Statistical Analysis

The value of the risk parameter was calculated using Kaplan-Meier survival curves. To see whether the risk factors were independent of clinical variables including age, gender, grade, and mutation, researchers used multivariate cox analysis and stratified data analysis. *p* < 0.05 was found statistically important. For all statistical studies, R software (v3.6.1) was utilized.

## Results

### Identification of Exercise-Related Gene TLR1 of PPI Network

**FIGURE 2:**
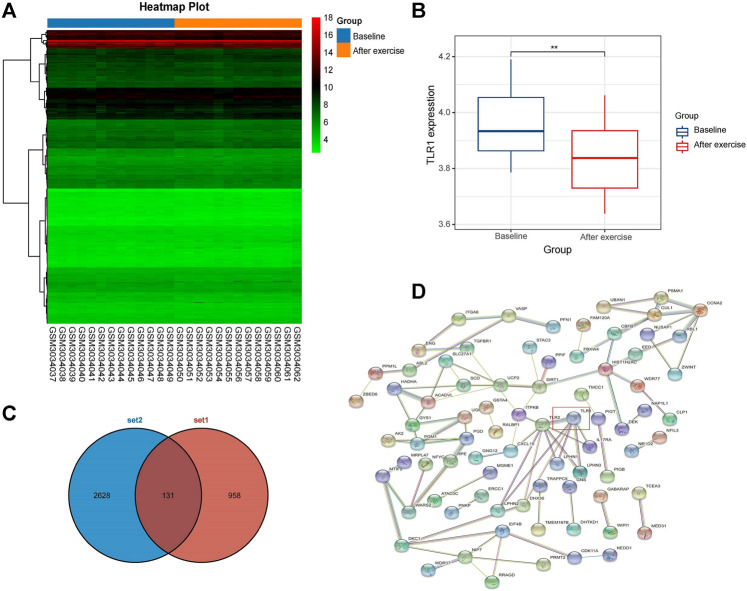
**(A)** DEGs hierarchical cluster analysis between baseline and post training groups in GSE111551 database. 958 DEGs related to exercise were identified. Red, up-regulated genes, blue, down-regulated genes **(B)** The expression of TLR1 in exercise group **(C)** Venn map of two gene sets **(D)** 131 exercise-related DEGs were included in PPI network. The nodes indicated proteins. The edges represented protein’s interaction.

Twenty-six transcriptome data subsets were collected from the GSE111551 database. The database was used to examine the baseline and exercise groups’ mRNA expression levels. There were 958 differentially expressed genes (DEGs) between the baseline and after exercise groups (Figure 2A). TLR1 was found to be down-regulated among them. Then we further made the difference of TLR1 between the two groups. Wilcox test study revealed a large differential between the two classes, with TLR1 expression decreasing dramatically in the exercise community (Figure 2B). Then we selected 2,628 survival differentially expressed genes from TCGA-LGG database and 131 differentially expressed genes from the intersection of the two genes (Figure 2C). Using the String database, a PPI network of differentially expressed genes was developed. 131 nodes are included in this network and may play a crucial role in aerobic exercise (Figure 2D). The first 10 genes were chosen by Cytohubba plugin (Cao et al., 2018) (Supplementary Figure S1) and sequentially ordered as follow: TLR1, SIRT1, CCNA2, HIST1H2AC, EED, SCD, ELAVL1, ACADVL, NIP7, UCP2. We selected the gene TLR1 at the core of the network.

### TLR1 Expression and Methylation in Low-Grade Glioma

**FIGURE 3:**
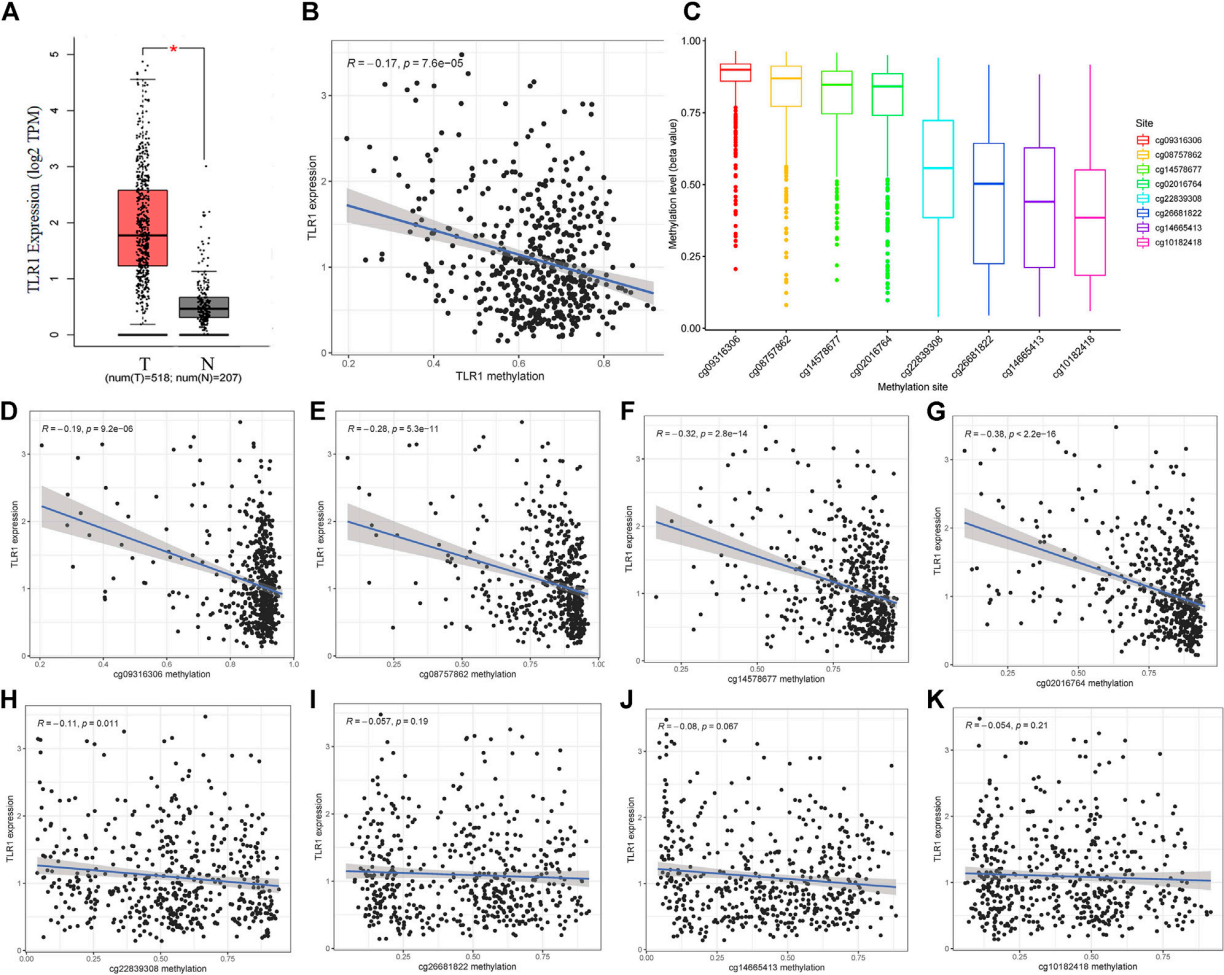
The expression and methylation of TLR1 in LGG tissues and normal tissues revealed by bioinformatic analysis. **(A)** TLR1 mRNA is strongly expressed in LGG tissues in TCGA dataset. **(B)** The expression of TLR1 was negatively regulated by DNA methylation. **(C)** The distribution of 8 TLR1 DNA promoter CpG sites. **(D–K)** Correlation analysis ofTLR1 CpG sites and TLR1 mRNA expression.

With GEPIA, we studied the RNA-sequencing results of 518 LGG tissues from TCGA and 207 normal samples from the GTEx project and discovered that TLR1 mRNA was strongly expressed in LGG tissues but not in normal tissues (Figure 3A). We found a negative association (*r* = 0.17, *p* < 0.0001) between TLR1 expression and TLR1 DNA methylation, as seen in (Figure 3B). Figure 3C clearly shows the distribution of eight TLR1 CpG locations. Then, using Pearson correlation analysis, the TLR1 CpG sites where methylation was highest closely associated with TLR1 mRNA expression were identified (Figures 3D–K).

### The Therapeutic and Prognostic Importance of TLR1 Expression According to TCGA Site

**FIGURE 4:**
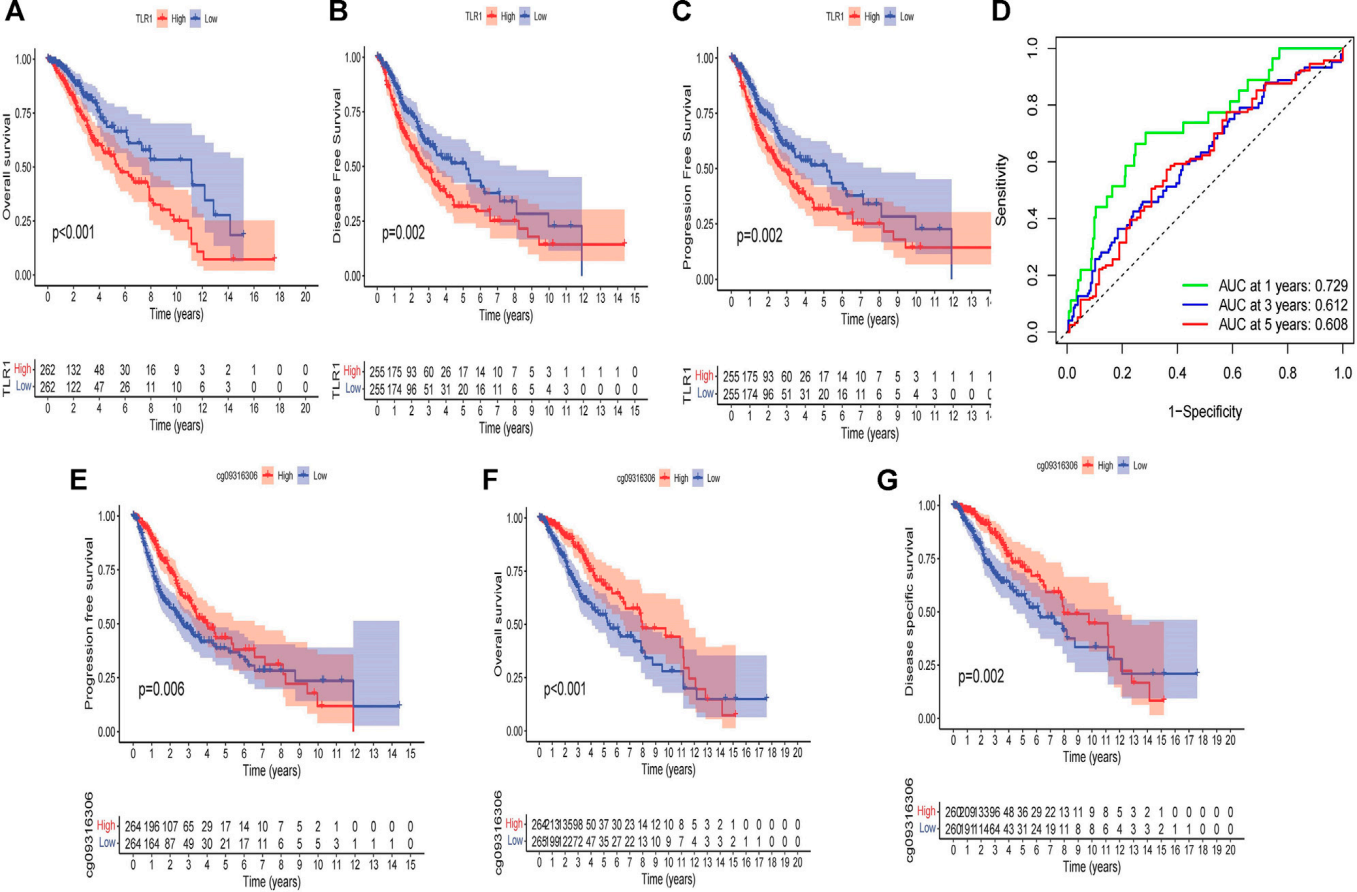
Kaplan-Meier curves. **(A)** over survival in all patients, grouped by low and high TLR1 expression. **(B)** disease-free survival in all patients, grouped by low and high TLR1 expression. **(C)** progression free survival in all patients, grouped by low and high TLR1 expression. **(D)** AUC values for predicting LGG survival rates at 1, 3, and 5 years. **(E)** progression free survival in all patients, grouped by low and high methylation level of cg09316306 site. **(F)** over survival in all patients, grouped by low and high methylation level of cg09316306 site. **(G)** disease-specific survival in all patients, grouped by low and high methylation level of cg09316306 site.

According to a Kaplan-Meier survival study, LGG patients with elevated TLR1 expression had a lower overall survival (OS) and disease-free survival (DFS) period (Figures 4A,B). Furthermore, patients with elevated TLR1 expression had shorter progression free survival (PFS) than those with poor TLR1 expression (Figure 4C). Taking cg09316306 as an example, elevated amounts of methylation at the selected CpG sites were also correlated with a stronger OS and PFS in LGG patients, according to Kaplan-Meier plots (Figures 4D–F). The relationship between other CpG sites and survival can be seen in Supplementary Figure S2. The LGG patients in the TCGA were then classified into low and high subgroups based on the median TLR1 expression or methylation value. The chi-square test was used to investigate the specific association of TLR1 expression and methylation with a set of clinical characteristics. TLR1 expression was found to be highly associated with age (*p* = 0.0263) and grade (*p* < 0.0001), as seen in Table 1. Similarly, age (*p* = 0.0061), grade (*p* < 0.0001) and TLR1 expression (*p* = 0.0166) had an impact on the degree of TLR1 methylation.

**TABLE 1:** Correlation between TLR1 mRNA expression/methylation and clinicopathologic features in TCGA database.

Covariates	Type	Total	ARID5A expression		P value	ARID5A methylation		P value
			High	Low		High	Low	
Age	<40	260 (49.34%)	143 (54.37%)	117 (44.32%)	0.0263	146 (55.51%)	114 (43.18%)	0.0061
	≥40	267 (50.66%)	120 (45.63%)	147 (55.68%)		117 (44.49%)	150 (56.82%)	
Grade	G2	260 (49.34%)	102 (38.78%)	158 (59.85%)	<0.0001	158 (60.08%)	102 (38.64%)	<0.0001
	G3	267 (50.66%)	161 (61.22%)	106 (40.15%)		105 (39.92%)	162 (61.36%)	
Gender	female	239 (45.35%)	109 (41.44%)	130 (49.24%)	0.0872	111 (42.21%)	128 (48.48%)	0.1737
	male	288 (54.65%)	154 (58.56%)	134 (50.76%)		152 (57.79%)	136 (51.52%)	
expression	High	263 (49.91%)	263 (100%)	0 (0%)	0	117 (44.49%)	146 (55.3%)	0.0166
	Low	264 (50.09%)	0 (0%)	264 (100%)		146 (55.51%)	118 (44.7%)	
methylation	High	263 (49.91%)	117 (44.49%)	146 (55.3%)	0.0166	263 (100%)	0 (0%)	0
	Low	264 (50.09%)	146 (55.51%)	118 (44.7%)		0 (0%)	264 (100%)	

### TLR1’s Prognostic Significance Validated in Low-Grade Glioma From CGGA

**FIGURE 5:**
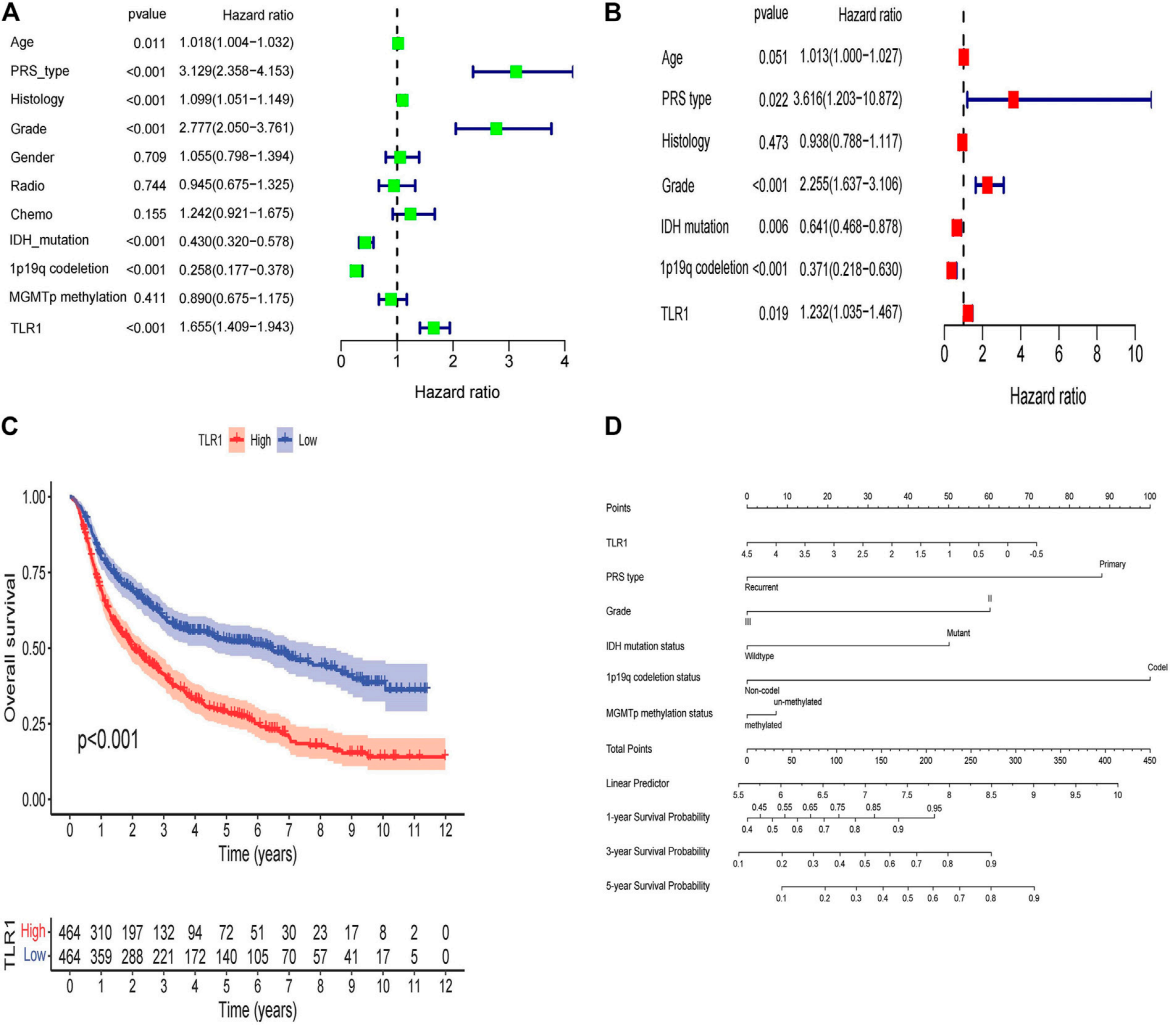
TLR1’s prognostic significance validated in LGG from CGGA. **(A)** The univariate and **(B)** multivariate Cox regression in LGG from CGGA. **(C)** TLR1 expression and overall survival in LGG patients in CGGA cohort. **(D)** Nomogram for predicting 1-, 3-, and 5-years OS of LGG patients in the CGGA cohort.

To confirm the prognostic importance of TLR1, we downloaded RNA-seq details and clinical data of 1,008 patients through CGGA. To validate TLR1’s prognostic importance, we used Kaplan-Meier research, which revealed that high TLR1 expression predicted low OS. We used univariate and multivariate Cox regression analysis to further validate TLR1’s independent prognostic significance (Figures 5A,B). TLR1, as seen in the forest diagram, maybe an independent prognostic biomarker (*p* = 0.019, hazard ratio = 1.232 (1.035–1.467)). In CGGA, PRS type (*p* = 0.022, hazard ratio = 3.616 (1.203–10.872), grade (*p* < 0.001, hazard ratio = 2.255 (1.637–3.106), 1p19q codeletion (*p* < 0.001, hazard ratio = 0.371 (0.218–0.630), and IDH mutation (*p* = 0.006, hazard ratio = 0.641 (0.468–0.878) could all be separate prognostic variables. According to a Kaplan-Meier survival study, LGG patients with elevated TRL1 expression had shorter overall survival (OS) (Figure 5C).

We created a nomogram to develop a quantitative tool for LGG prognosis (Figure 5D). Specific variables were assigned points using the point scale in the nomogram based on the multivariate Cox study. We quantify the cumulative points for each patient by summing the points of all variables and normalizing them to distribution of 0–100 by drawing a horizontal line to decide the value of each variable. By drawing a longitudinal line between the complete point axis and each prognosis axis, we may quantify the average survival rates for LGG patients at 1, 3, and 5 years, which can aid appropriate clinicians in developing clinical decision making for LGG patients.

### Examining TLR1’s Interactions With Immune Cell Infiltration

**FIGURE 6:**
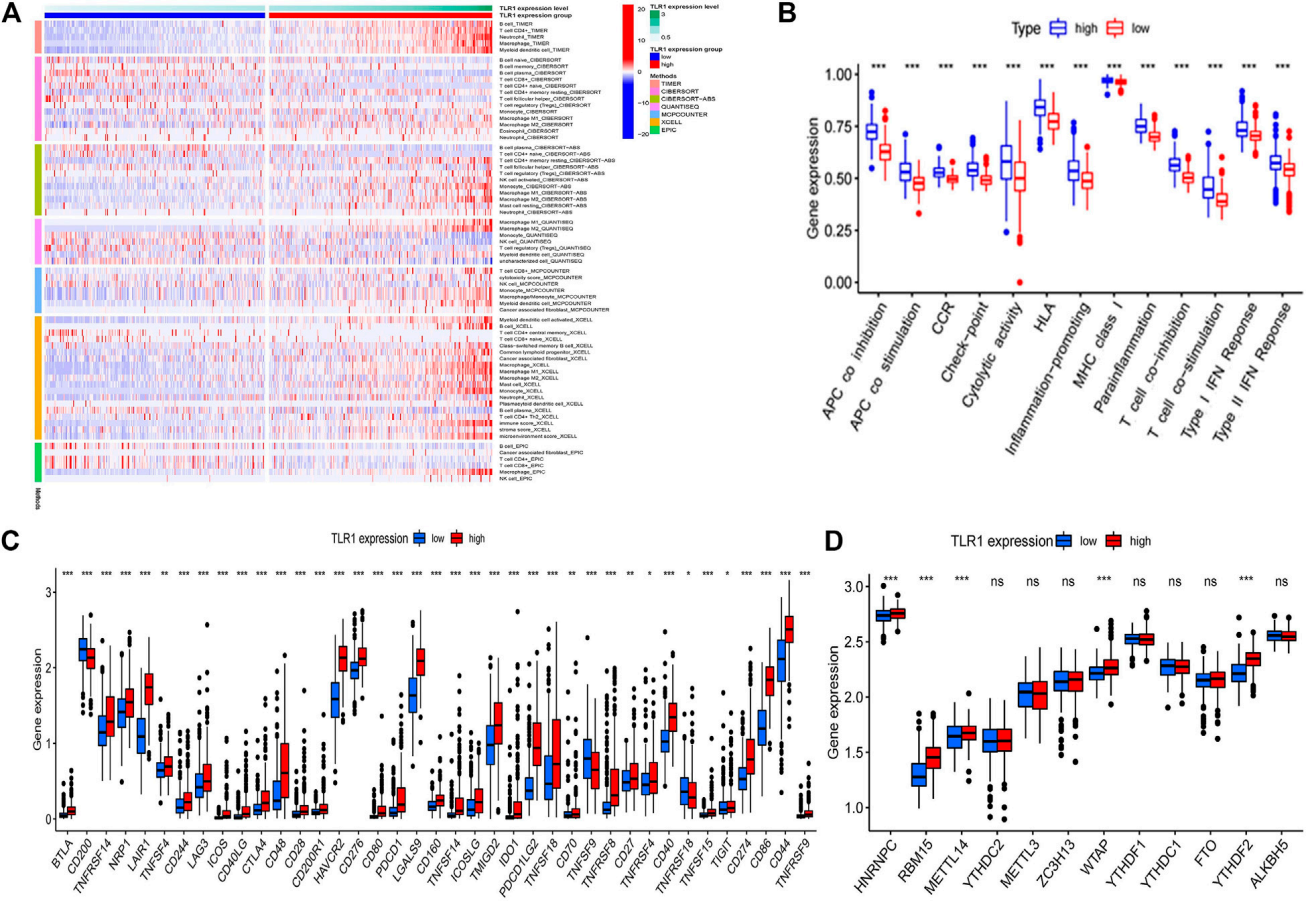
The Correlation of TLR1 expression with immune infiltration level in LGG. **(A)** the heatmap of immune responses based on different algorithms. **(B)** ssGSEA for the association between immune cell subpopulations and related functions. **(C)** immune checkpoints expression among the high and low groups. **(D)** The expression of m6A-related genes between high and low TLR1 risk group.

Figure 6A shows a heatmap of immunological responses depending on the algorithms. According to ssGSEA of TCGA-LGG data, correlation analysis of immune cell subtypes and associated activities showed that T cell functions such as cytolytic, check-point, MHC class1, co-inhibition, and co-stimulation were substantially different between the two groups Figure 6B. Because checkpoint inhibitor-based immunotherapies are so important, we looked at the differences in immune checkpoint expression between the two groups. Between the two groups of patients, we discovered a significant variation in the expression of PDCD1, CTLA4, and BTLA (Figure 6C). There were significant variations in the expression of 5 methyltransferases such as HNRNPC, METTL14, and RMB15 when the m^6^A expression-related mRNA was compared between the two groups (Figure 6D).

### Interaction Between TLR1 Methylation Site and Immune Cell Infiltration

**FIGURE 7:**
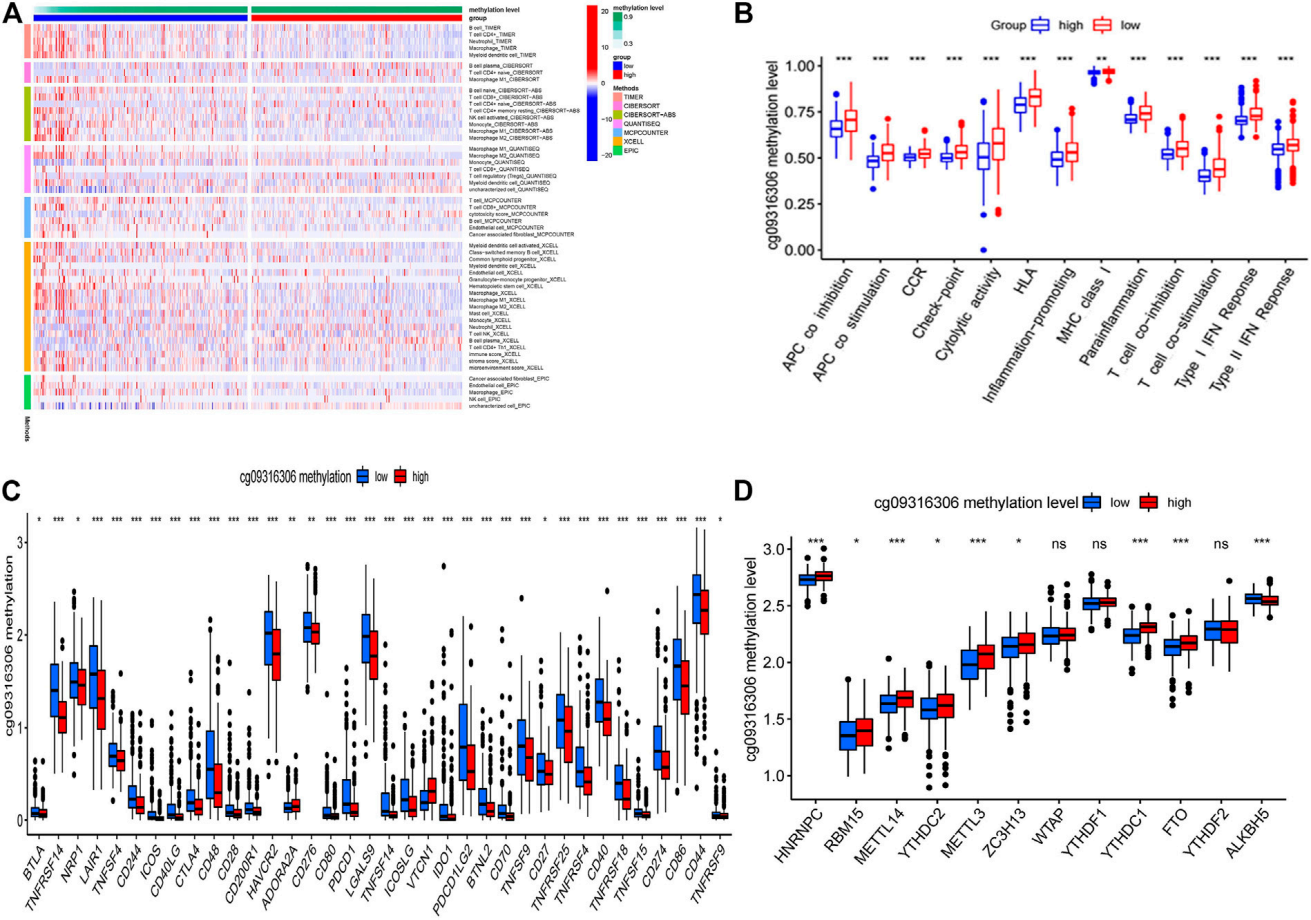
The Correlation of TLR1 methylation site cg09316306 and immune cell infiltration. **(A)** the heatmap of immune responses based on different algorithms. **(B)** ssGSEA for the association between immune cell subpopulations and related functions. **(C)** immune checkpoints expression among the high and low groups. cg09316306 site methylation risk groups. **(D)** The expression of m6A-related genes between high and low cg09316306 site methylation risk group.

The methylation site cg09316306 was taken as an example. Figure 7A shows a heatmap of immunological responses depending on the algorithms. According to ssGSEA, correlation analysis of immune cell subtypes and associated activities showed that T cell functions such as cytolytic, check-point, MHC class1, co-inhibition, and co-stimulation were all substantially different between the two groups (Figure 7B). We then looked at the differences in immune checkpoint expression between the two groups. Between the two groups of patients, we discovered a significant variation in the expression of PDCD1, CTLA4, BTLA, and VTCN1 (Figure 7C). There were significant variations in the expression of 9 methyltransferases such as HNRNPC, METTL14, and RMB15 when the m^6^A expression-related mRNA was compared between the high and low methylation levels groups (Figure 7D). Similarly, we also analyzed the relationship between the remaining 7 CpG sites and immune invasion. The results showed that 3 methylation CpG sites had no significant correlation with immune checkpoint (Supplementary Figure S3).

### Analysis of TLR1-Related Biological Processes

**FIGURE 8:**
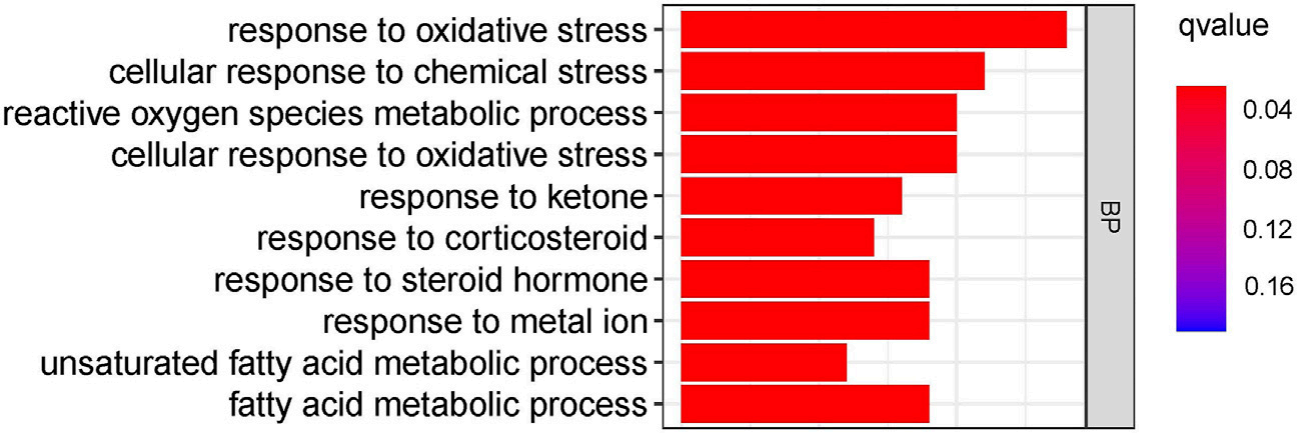
GO enrichment analysis of TLR1-related biological pathways in glioma.

GO enrichment analysis revealed that TLR1 is involved in pathways of oxidative stress, chemical stress, reactive oxygen species metabolic process, cellular response to oxidative stress, response to ketone, response to corticosteroid, response to a steroid hormone, response to the metal ion, unsaturated fatty acid metabolic process and fatty acid metabolic process among others (Figure 8). It is worth noting that in these numerous biological processes, Oxidative stress can cause DNA base changes, strand breaks, increased expression of proto-oncogenes, and inactivation of tumor suppressor genes. It is linked to the onset and progression of numerous cancers.

### Knockdown of TLR1 Suppresses the Malignant Phenotype of Glioma *in vitro*

**FIGURE 9:**
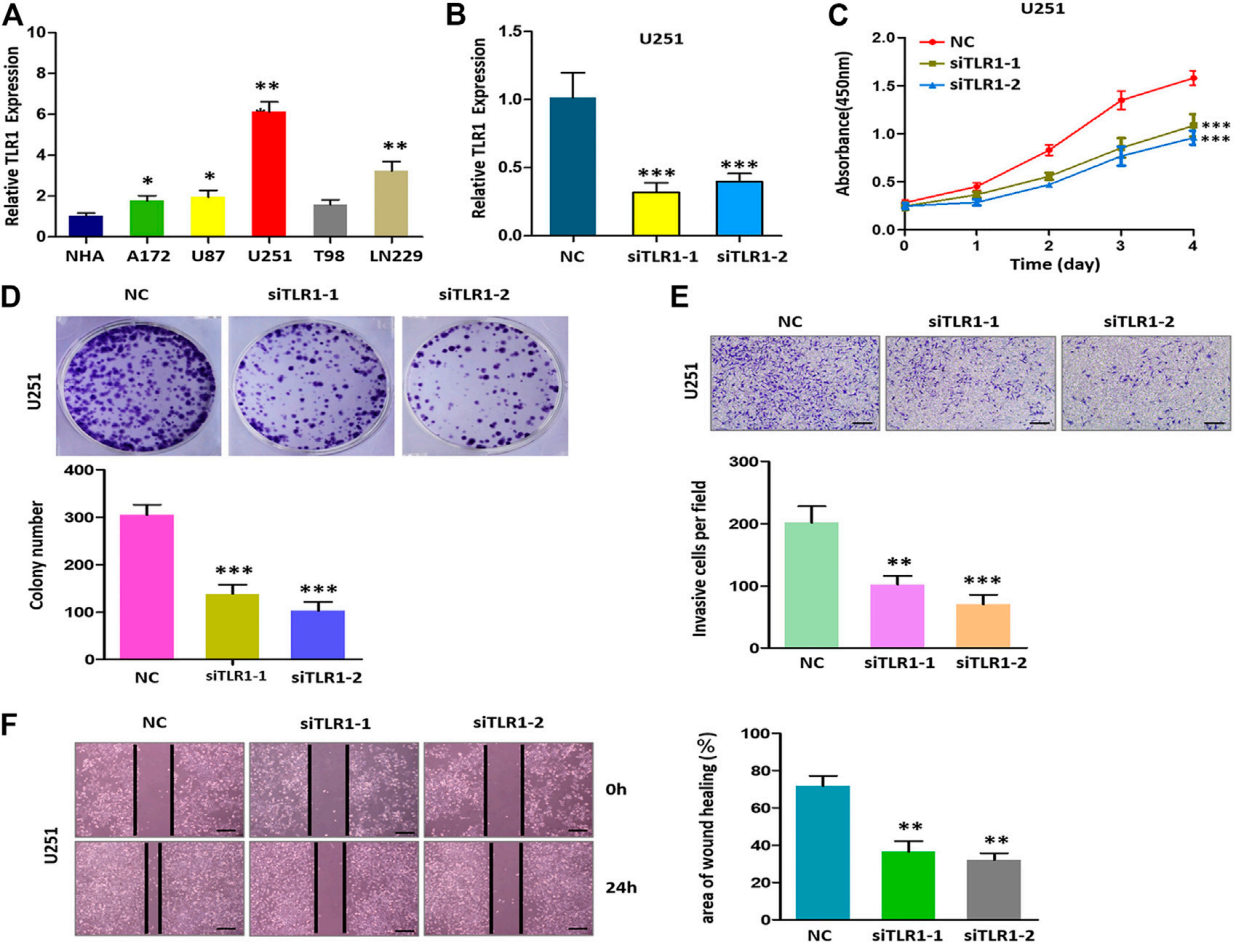
Decreased the expression of TLR1 inhibits proliferation, invasion, and migration of glioma cells *in vitro*. **(A)** Relative expression of TLR1 in 6 cell lines. **(B)** qRT-PCR to detect the relative silencing levels of TLR1 in the U251 cell line. **(C)** The CCK-8 assay was applied to detect the efficiency of TLR1 knockdown on the proliferation of the U251 cell line. **(D)** Images of the colony formation assay after knockdown of TLR1 in U251 cell line. **(E)** Images of the transwell assay results after knockdown of TLR1 in U251 cell line. **(F)** Representational images of the wound healing assay.

To explore the role of TLR1 in glioma, the expression of TLR1 in the six glioma cell lines (NHA, A172, U87, U251, T98, LN229) was analyzed respectively. The expression of TLR1 in the U251 cell line was higher than in other cell lines (Figure 9A). Then, the U251 cell line was selected for functional analysis. Using qRT-PCR, we have detected the efficiency of TLR1-siRNA (Figure 9B). The results of colony formation and CCK8 experiments demonstrated that lower expression of TLR1 significantly inhibited the ability of proliferation and colony formation of U251 cells (Figures 9C,D). The results of the transwell experiment indicated that the U251 cells exhibited significantly decreased invasion ability upon TLR1 knockdown (Figure 9E). The results of the wound healing experiment indicated that knockdown of TLR1 significantly inhibited the migration of U251 cells (Figure 9F). We measured the expression of PDCD1, CTLA4, BTLA and methyltransferases HNRNPC, METTL14 and RMB 15 in U251 cells with and without siTLR1-1, respectively. We found that the expression of six proteins in siTLR1-1 decreased (Supplementary Figure S4). These findings showed that TLR1 may be a glioma promoter. To uncover the underlying processes, further study is required.

## Discussion

Medium-intensity and rhythmic activity, which is marked by low intensity, speed, and long length, is referred to as aerobic exercise (Dahamsheh et al., 2019). The GEO data set selected in this study adopts the way of running. This type of exercise uses oxygen to fully burn sugar in the body while still consuming body fat, improving cardiopulmonary function, preventing osteoporosis, and regulating psychological and mental state (Santos et al., 2017). Epidemiological data show that exercise can prevent the occurrence of some cancers and reduce the risk of disease recurrence (Schmidt et al., 2015), which promotes extensive research on exercise intervention for cancer patients.

Epigenetics is the study of how non-gene sequence changes affect gene expression levels (Zhang et al., 2016). Methylation is the most stable epigenetic mechanism, and epigenetic modification also plays an important role in exercise adaptation. A large number of studies have confirmed the interaction between exercise and epigenetics (Liang et al., 2021). Recently, more and more attention has been paid to the application of exercise in tumor diseases. We found the difference gene TLR1 between baseline group and post training group in the data set, and TLR1 was differentially expressed in low-grade glioma and normal population in TCGA data set. In LGG tissues, TLR1 mRNA expression was negatively associated with the methylation of TLR1. We discovered that TLR1 expression and methylation were linked to several important characteristics, such as histological and molecular forms. The importance of TLR1 hypermethylation and low expression in LGG patient’s positive prognosis was supported by a Cox regression model.

A significant number of studies have shown that irregular DNA methylation is essential in the onset and development of LGG (Mathur et al., 2020). In this study, we confirmed that TLR1 methylation status affects the expression of TLR1mRNA. TLR1 methylation was negatively correlated with TLR1 mRNA expression in LGG tissues. TLR1’s low expression in LGG tissues may be explained by this negative association. Then, we found The methylation of five CpG sites in the DNA promoter was closely related to TLR1 mRNA expression. We also looked into the function of TLR1 DNA methylation and CpG sites locus in prognosis. We found that hypermethylation of TLR1 at this locus was corrected with good OS and improved PFS in LGG, and we further verified it by multivariate regression. As a result of the methylation of CpG sites, TLR1 is negatively controlled, and the methylation status of TLR1 can be an important predictor of OS and PFS.

Several genes linked to glioma pathogenesis have been discovered, but the prognostic and clinical importance of the TLR1 gene in glioma remains uncertain. In the report of the TLR1 gene, elevated expression of TLR1 is corrected with a good prognosis of pancreatic cancer (Lanki et al., 2019), but it has never been studied in LGG. In this analysis, we used the TCGA database to investigate the connection between low TLR1 expression and survival rates, and we double-checked the findings using the CGGA database. Encouragingly, in LGG patients, low TLR1 expression is highly associated with improved OS than elevated TLR1 expression. Multivariate Cox regression further confirmed this conclusion, suggesting that decreased TLR1 expression is a powerful prognostic factor for OS in LGG patients. TLR1 is a successful biomarker for assessing the prognosis of LGG patients, according to our findings.

One of the most frequent causes of cancer development is chronic local inflammation, which may be caused by a change in the microenvironment (Shacter and Weitzman, 2002). The function of local inflammation is complex, because some features are conducive to tumor progression, while others can prevent tumor progression (Schulz et al., 2019). The relationship between tumors and inflammation has always been a concern. In recent years, more and more researchers use experimental data to show that tumor-associated inflammation can promote tumor growth and progress by promoting angiogenesis and metastasis, subverting anti-tumor immune response, and changing the sensitivity of tumor cells to chemotherapy drugs (Weenink et al., 2019). Persistent and uncontrollable inflammatory microenvironments can also trigger gene mutation and lead to tumorigenesis (Chauhan and Trivedi, 2020). TLRs, or toll-like receptors, are important mediators of local inflammation. They identify foreign molecules and activate the NF-B pathway, causing inflammatory cytokines and interferon-gamma to be released (Li et al., 2010; O'Neill et al., 2013). The finding that TLR1 expression correlates with immune cell markers suggests that TLR1 can regulate tumor immunity in LGG. In addition, we found that 5 of the 8 methylation sites were closely related to the immune checkpoint. At the same time, it opens a new way for the immunotherapy of LGG. Furthermore, reducing TLR1 expression substantially slowed the cell cycle and decreased LGG cell proliferation, emigration, and infiltration *in vitro*.

Our review has certain inherent limits. First of all, because there are relatively few studies on exercise in cancer patients, we screened the exercise genes from normal people. Second, we were unable to establish the predictive role of TLR1 methylation in LGG owing to the limitations of the CGGA database. Third, the TCGA database is the only one that contains PFS content, and the connection between TLR1 expression and PFS cannot be checked in other databases. Finally, while enrichment analysis enabled us to investigate the biological process of TLR1 in gliomas, the comprehensive mechanism of linking TLR1 expression and TLR1 methylation with LGG development needs additional biomedical research. The recent findings, on the other hand, are encouraging and call for further research into discovering potential prognostic biomarkers of LGG.

## Data Availability

The original contributions presented in the study are included in the article/Supplementary Material, further inquiries can be directed to the corresponding authors.
