# Supplementary figures and images for: Methylation and Expression of the Exercise-Related TLR1 Gene Is Associated With Low Grade Glioma Prognosis and Outcome
Source: Front Mol Biosci. 2021 Nov 16;8:747933. doi: 10.3389/fmolb.2021.747933 (PMC8635206; doi:10.3389/fmolb.2021.747933)

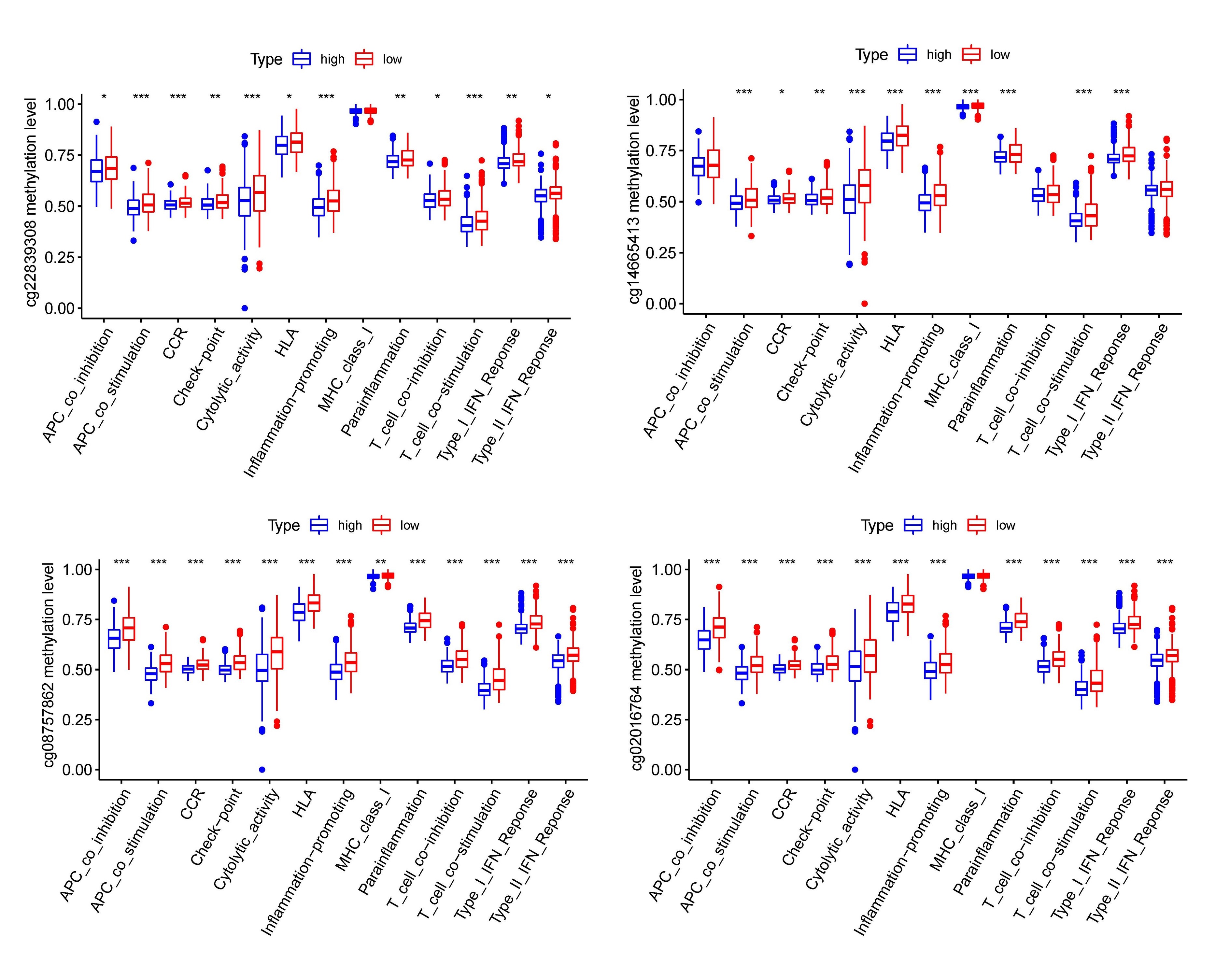

Supplement: Supplementary file 1 [file Image3.JPEG]

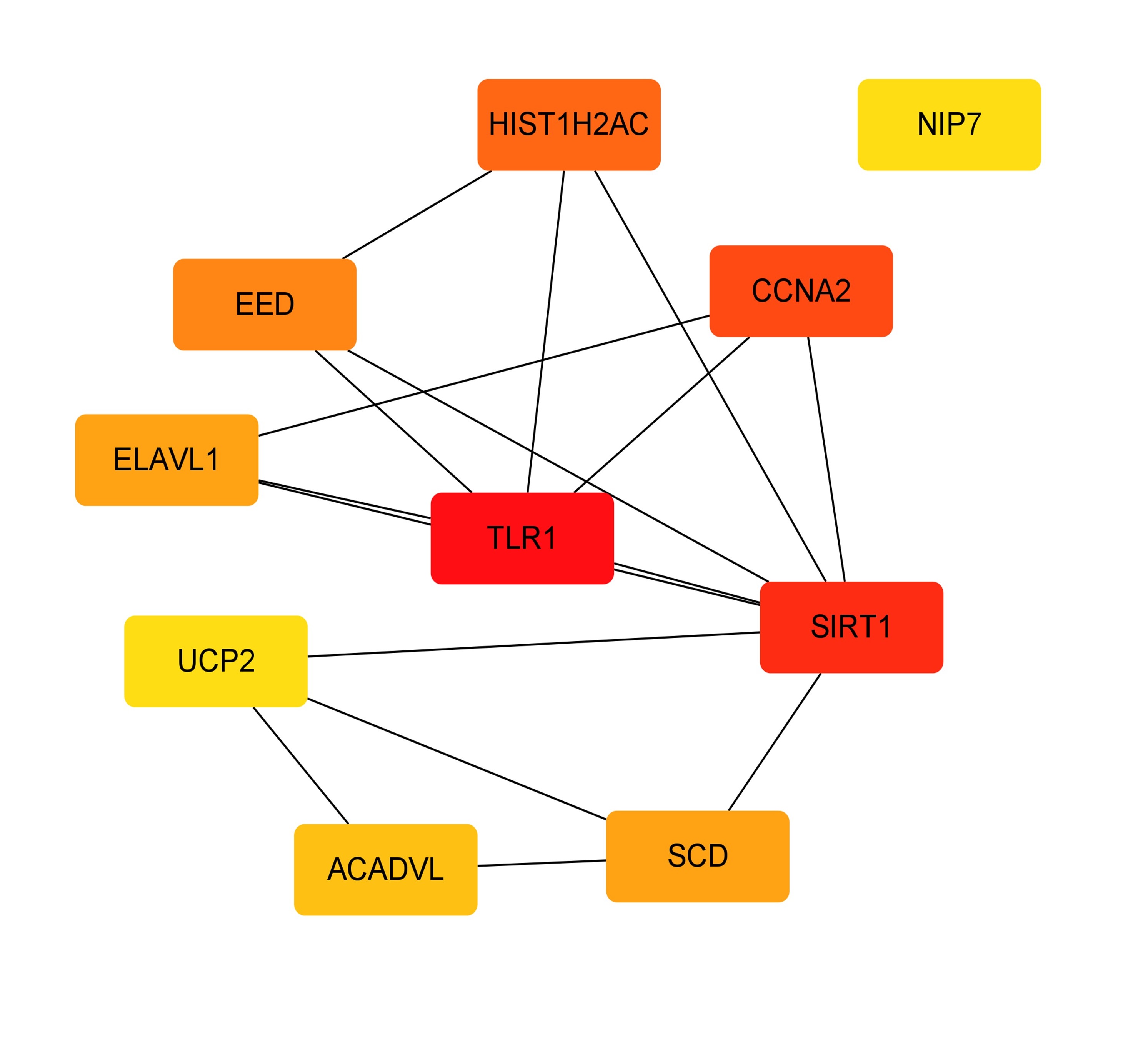

Supplement: Supplementary file 2 [file Image1.JPEG]

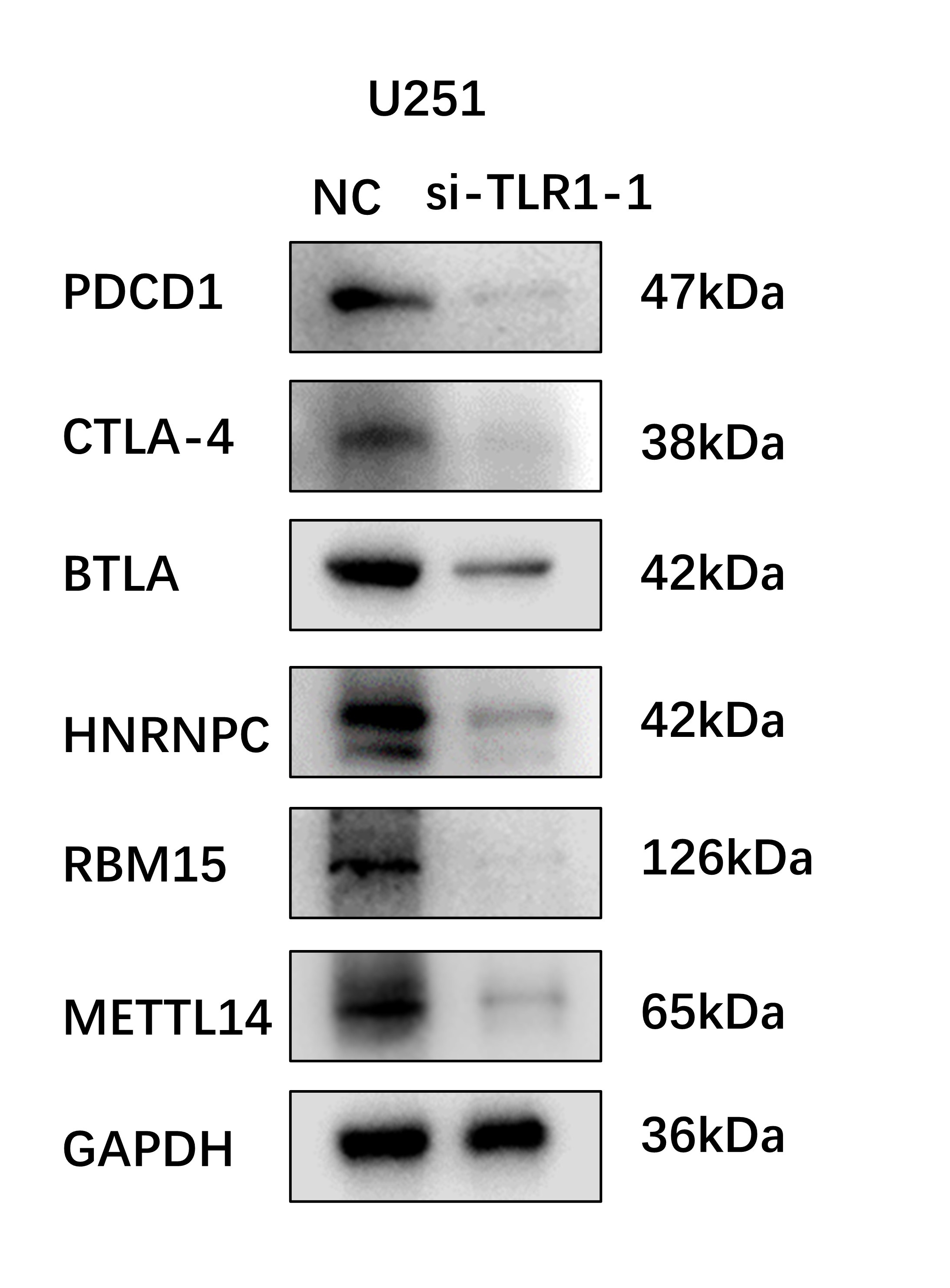

Supplement: Supplementary file 3 [file Image4.JPEG]

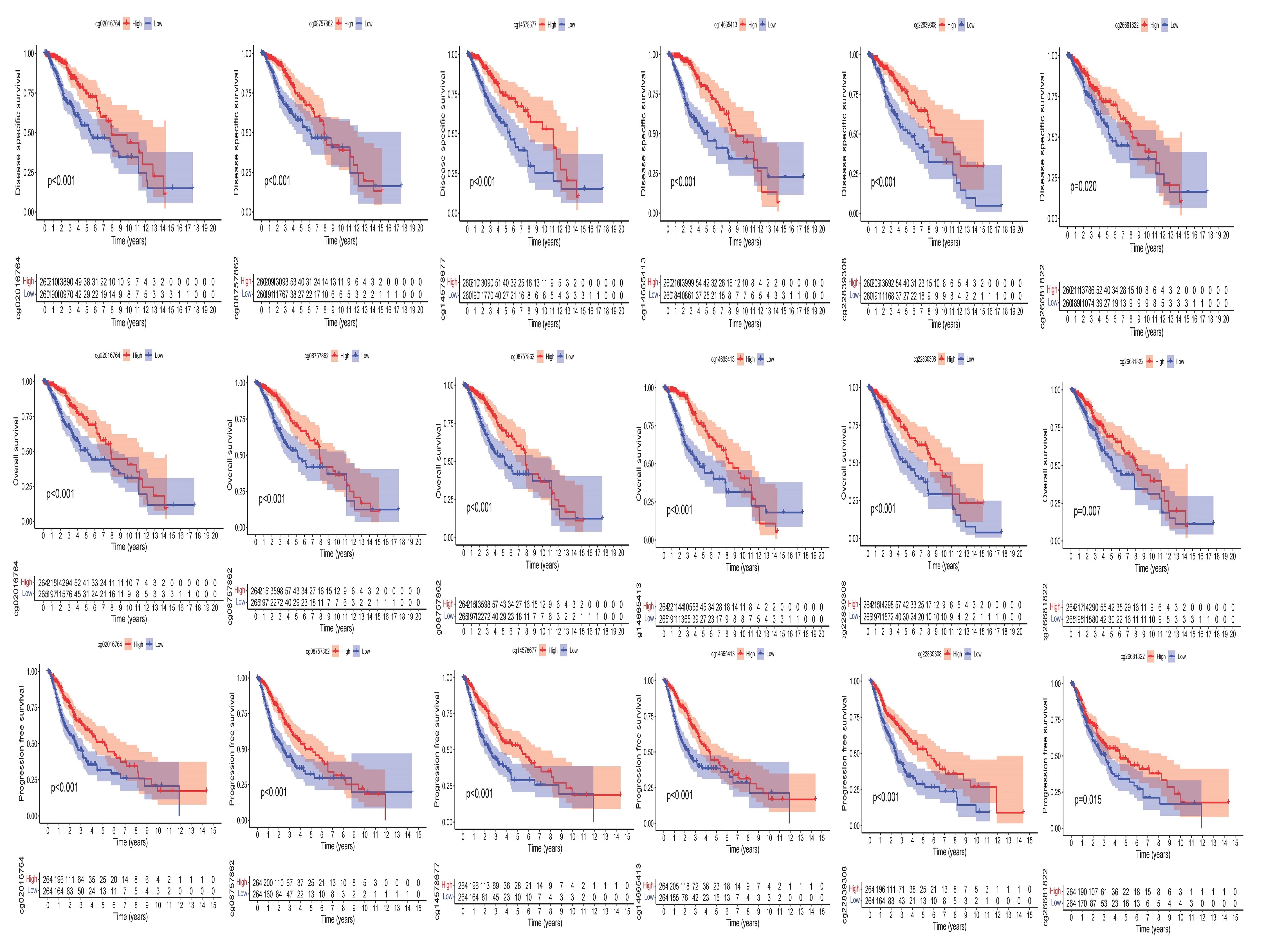

Supplement: Supplementary file 4 [file Image2.JPEG]
